# The Enigma of Sponge-Derived Terpenoid Isothiocyanate–Thiocyanate Pairs: A Biosynthetic Proposal

**DOI:** 10.3390/md23050220

**Published:** 2025-05-21

**Authors:** Tadeusz F. Molinski

**Affiliations:** 1 Department of Chemistry and Biochemistry, MC3568, University of California, 9500 Gilman Drive, La Jolla, San Diego, CA 92093, USA; tmolinski@ucsd.edu; 2 Skaggs School of Pharmacy and Pharmaceutical Sciences, University of California, 9500 Gilman Drive, La Jolla, San Diego, CA 92093, USA

**Keywords:** alkyl isothiocyanate, alkyl thiocyanate, farnesyl pyrophosphate, geranylgeranyl pyrophosphate, malaria, marine natural product, nudibranch, Porifera, isonitrile, rhodanese, thiocyanate

## Abstract

The co-occurrence of rare terpenoid thiocyanates (R-SCN), structurally similar to their more common isothiocyanate isomers (R-NCS), poses an enigma: how does the accepted path, terpenyl cation R^+^ → R-NC → R-NCS, accommodate R-SCN? The mystery can now be rationalized by the consideration of three biosynthetic motifs: terpenoid carbocation (R^+^) capture by cyanoformate, NC-COOH (itself in equilibrium with NC^−^ and CO_2_); co-localized rhodanese (a dual-function enzyme) that can both convert fugitive inorganic NC^−^ to thiocyanate ion, NCS^−^, and alkyl isonitriles to alkyl isothiocyanate (R-NC → R-NCS) and adventitious capture of the NCS^−^ by R^+^. The former two scenarios explain the preponderance of isothiocyanates, R-NCS, as products of a linear reaction path—the α-addition of S^0^ to R-NC—and the third scenario explains minor, less stable thiocyanates, R-SCN, as products of the adventitious capture of liberated NCS^−^ by the penultimate R^+^ precursor. DFT calculations support this proposal and eliminate other possibilities, e.g., the isomerization of R-NCS to R-SCN.

## 1. Introduction

**Figure 1:**
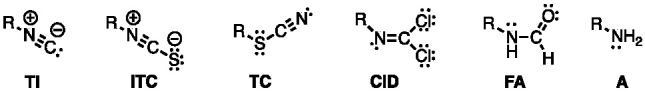
Lewis structures (closed-shell) of terpene isonitriles (**TI**s), ‘isonitriloids’–isothiocyanates (**ITC**s), thiocyanates (**TC**s), carbonimidic dichlorides (**CID**s), and products of reaction with H_2_O formamides (**FA**s) and amines (**A**s).

**Figure 2:**
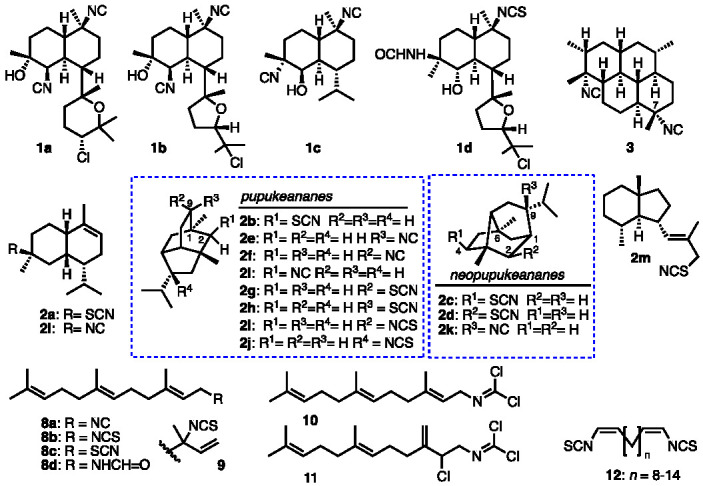
Terpenoid isonitriles (**TI**); kalihinols A (**1a**), B (**1b**), M (**1d**), and synthetic MED6-189 (**1c**); 7,20-diisocyanoadociane (**3**); and related isothiocyanates (**ITC**s, e.g., **1d**, **2i**–**j**, **8b** and thiocyanates (**TC**s), **2a**–**d**, **g**–**k**, **m**. *bis*-Isothiocyanate **12** is a non-terpenoid.

Exotic terpenoid isonitriles (**TI**s, Figure 1), which occur exclusively within the domain of certain genera of marine sponges (Porifera), e.g., *Acanthella*, *Adocia*, *Axinella*, *Axynissa*, *Cymbastella*, among others, and the sea slugs (nudibranchs) that prey upon them have been known since the early 1970s. Marine isonitriles and their derivatives have been extensively reviewed [1,2,3]. Examples of cyclized terpenoid isonitriles and their accompanying α-adducts (**1a**–**c**, **2a**–**l**, **3**) are depicted in Figure 2.

Only recently have certain members (e.g., 7,20-diisocyanoadociane (**3**) [4] and kahilinols A (**1a**) [5] and B (**1b**) [6]) been identified and investigated as potential antimalarial therapeutics. The natural product **1b** exhibits potent activity against the chloroquine-resistant plasmodium Dd2 (IC_50_ = 4.6 nM). MED6-189 (**1c**), a synthetic analog developed from **1b**, shows promising in vitro antiplasmodial activity (Dd2 IC_50_ = 47 ± 7 nM) and strong efficacy in plasmodium-infected mice, with no apparent toxicity or hemolytic liabilities [7].

**TI**s are often accompanied by the corresponding products of α-addition to the triple -NC bond: amides, carbonimidic dichlorides (**CID**s), and, most commonly, alkyl isothiocyanates (**ITC**s) (Figure 1) [8]. Sponge-derived isonitrile biosynthesis can be rationalized by the capture of late-stage terpenoid carbocations arising from activated precursors (GPP, FPP, and GGPP) that promote C-C cyclizations, Wagner–Meerwein rearrangements, and a final capture by a biosynthetic ‘NC vector’ [9] to generate isonitriles, R-NC. Isothiocyanates, R-NCS, e.g., kalihinol M (**1d** [10]) (Figure 2), and **2i**, **j**, **k**, follow as the products of sulfur transfer reactions from low-valent S donors (e.g., thiosulfate, etc.) to R-NC. The terpenoid carbon backbones of **TI**s and related derivatives, including isothiocyanates (**ITC**, Figure 1), occur in limited plant species, but **TI**s are synthesized only by sponges. For example, the common plant sesquiterpene (C_15_) skeletons of amorphane, drimane, and bisabolane are all represented in the sponge **TI**, **ITC**, and **TC** family, but sponges and their predators (nudibranchs) are the exclusive provenance of the carbon skeletons axinyssane [11,12], pupukeanane [13,14], neopupukeanane [15] and the diterpenes (C_20_) adociane [4], amphilectane [16,17], and isoneoamphilectane [18], among others.

The startling discovery of co-localized thiocyanates, **TC**s, (R-SCN), e.g., the *cis*-fused bicyclic 4-thiocyanato-9-cadinene **2a** [19] (more accurately, an amorphene, having the same carbon skeleton as the amorphene isonitrile **2l** [20]), and regioisomeric 2- and 4-thiocyanato*neo*pupukeananes (**2c**,**d**) [21,22] poses an enigma: ‘how can a linear biosynthetic pathway of R-NC → R-NCS accommodate R-SCN?’ In plants, the biosynthesis of allyl thiocyanate and its isomeric allyl isothiocyanate, and related pungent volatile natural products of mustard oil, horseradish, garlic, and many other Cruciferate, has long been understood [23], e.g., decomposition of the glucosinolate, sinigrin, derived from the amino acid, Met, but the origin of sponge-derived thiocyanates is less clear. Other unusual features present themselves. For example, the incipient precursor of **2c** and **2d** resembles the storied ‘non-classical’ norbornyl cation [24] where the positive charge is delocalized between the C2 and C4 positions which may explain the outcomes of *S*_N_: *exo* **TC** regioisomers isolated in almost equal proportions [21,22] with the same skeleton as 9-isocyano*neo*pupukeanane **2n** [15]. Rounding out the thiocyanates are (–)-cavernothiocyanate (**2m**) [25] and **2g**, **h** [26], which share the pupukeanane skeleton with isothiocyanates (R-NCS, e.g., **2i** [27], **j** [14].

Now this enigma is addressed by the consideration of the putative interplay of three integrated biosynthetic sequelae: the capture of the terpenoid carbocation R^+^ by cyanoformate, NC-COOH (**CF**, see below [9] (itself in equilibrium with NC^−^ and CO_2_ and derived from Gly), reactions promoted by co-localized rhodanese, a dual-function enzyme that can both convert inorganic NC^−^ to thiocyanate ion, NCS^−^ but also alkyl isonitriles to alkyl isothiocyanate (R-NC → R–NCS), and the promiscuous interception of terpenoid carbocations by co-liberated inorganic NCS^−^.

## 2. Results and Discussion

### 2.1. Chemistry and Bonding in TCs and ITCs

While hundreds of marine-derived **TI**s, **ITC**s, and **FA**s have been described [1], only seven **TC**s are known (Figure 2). The structures of the **TC**s have been examined on a case-by-case basis, with their isomeric or similar isothiocyanates (**ITC**s). The ‘reverse engineering’ of the likely terminal steps in their biosynthesis by probing the thermodynamics of bond breaking and bond forming provides insights into what could be possible and what is improbable. For the purposes of an informed discussion, a brief review of the chemistry of the cumulated-bond functional group, NCS, in **TC** and **ITC** molecules is in order. **TC** and **ITC** isomers are easily differentiated spectroscopically by ^13^C NMR and IR. **ITC** shows a very intense, broad stretching frequency at ~2100 cm^−1^, while the **TC** IR band is about the same frequency, but narrower and of medium intensity. The ^13^C NMR signal of C at the point of attachment reflects the difference in the electronegativity of S and N: **ITC** shows C-N_α_ of approximately *δ*~75, while the C-S_α_ signal in **TC** is *δ*~60 ppm [19].

**Figure 3:**
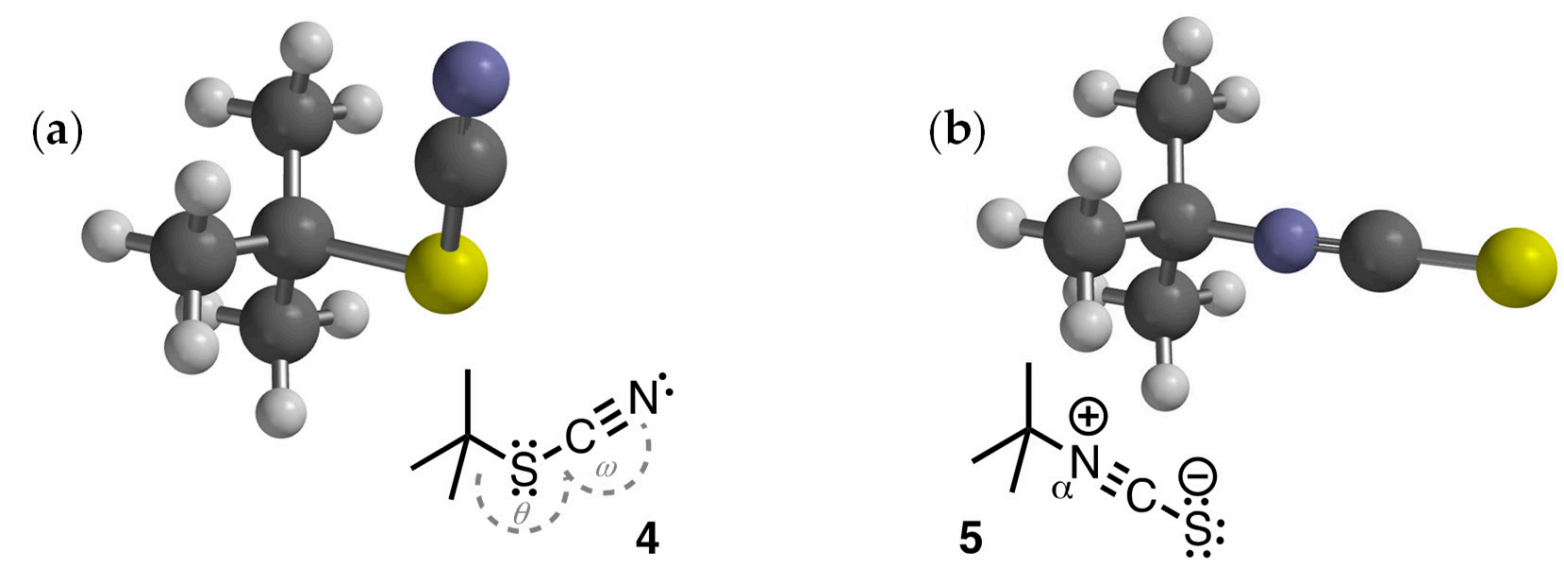
DFT-optimized molecular structures (ωB97X-D 6-31G*), dipole moments, key bond angles, and distances, *d*, of isomeric **TC** and **ITC**. (**a**) *t*-butylthiocyanate (**4**): µ = 4.48 D *θ*_thio_(C-S-C) = 100.0°, *d*_thio_(C–S) = 1.88 Å; and (**b**) *t*-butylisothiocyanate (**5**): µ = 6.56 D, *θ*_tiso_(C–N_α_–C) = 176.8°, *d*_iso_(C-N_α_) = 1.44 Å. The major resonance form (closed-shell Lewis structure) is depicted for each.

The isomers *t*-butylthiocyanate (**4**) and *t*-butylisothiocyanate (**5**) (Figure 3) serve as convenient models for comparisons with their terpenoid natural product counterparts. Bonding in the triatomic grouping NCS differs between **TCs** and **ITC**s. The DFT calculations of the optimized geometries and energies of each (Figure 1) show, as expected, that the geometry of N-C-S is linear due to sp hybridization at C (*ω* = 179.8°), consistent with the X-ray crystal structures of the natural products **ITC**s [14] and **TC**s 19]. The bonding of the heteroatom attached to the alkyl group C (notated, in this paper, as the α atom) differs significantly. The corresponding bond angles and bond lengths of **4** and **5** (*θ*(C-S-C) = 100° and (*θ*(C-N_α_-C) = 176.8°) reflect predominantly sp^3^ and sp hybridization, respectively, of the α-heteroatom attached to the *t*-Bu group. The shorter C-N_α_ bond length in **5** (*d*_2_ = 1.44 Å) is evident of a stronger σ bond between the *t*-Bu group and its α-heteroatom compared to **4** (*d*_1_ = 1.88 Å; see below, as expected, for row 2 versus row 3 elements) recapitulates the thermodynamic stability of **5** over **4** [19]. The DFT minimized structure of 1-isothiocyano-1-methylcyclohexane (**S1**) displays similar bond parameters. From the DFT-calculated energies of **4** and **5**, the latter is shown to be more stable (∆E = –14.8 kcal·mol^−1^; see Supporting Information), and the isomerization of **5** to **4** is not spontaneous.

**ITC**s are electrophilic: like isonitriles, they undergo addition reactions at the cumulated bond, even with weak nucleophiles. **TC**s reluctantly undergo *S*_N_2 substitution reactions at the attached C when 1° or 2°, but not 3°. Allylic **ITC**s and **TC**s both participate in sigmatropic [3] rearrangement (see below). Three scenarios that may account for the generation of **TC** natural products are considered.

### 2.2. Dissociation–Reassociation

The isomerization of **ITC** to **TC** was examined as a possible linear pathway linking the corresponding **TI** (R-NC → R–NCS → R-SCN). Necessarily, such an isomerization must follow a unimolecular rate law, *R* = *k*[R-NCS], and an *S*_N_1 mechanism due to the 2° or 3° alkyl group that is almost invariably the point of heteroatom attachment in **TI**, **ITC**, and **TC** natural products. The leaving group ability of the thiocyano group in R-SCN is at best weak, while in R-NCS, it is considerably poorer due to the stronger R-N bond. Experimentally, the kinetic outcome of S_N_ reactions with NCS^−^ favors the **TC** product [28].

**Figure 4:**
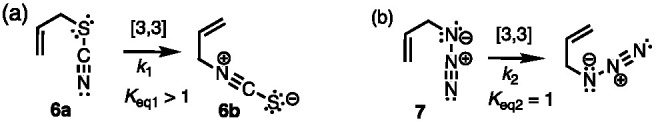
Isomerization of allylic isonitriloids, C_3_H_5_-NCS and C_3_H_5_-N_3_. (**a**) Sigmatropic [3] rearrangements: (**a**) allyl thiocyanate (**6a**) to allyl isothiocyanate (**6b**). (**b**) Degenerate rearrangement of allyl azide (**7**).

For various reasons, calculating the competitive rates of the *S*_N_ reaction of a row 2 element, N, with a row 3 element, S, is difficult, but we can easily obtain semi-quantitative estimates from the product distributions of sigmatropic [3] rearrangements of allylic thiocyanate (e.g., **6a**) to isothiocyanates (e.g., **6b**) and the degenerate rearrangement of isoelectronic allyl azide (**7**, Figure 4), where C-N/C-S bond-forming and bond-breaking reactions are simultaneous. While the reaction rates of the [3] rearrangement of allylic azides (Winstein rearrangement [29]) are fast (most rapidly isomerize at ambient temperatures [30]), CH_2_=CH-NCS and CH_2_=CH-SCN interchange more slowly [28]. For example, the isomerization of the latter to the former occurs upon distillation (bp 150-2 °C) [28]); at *K*_eq_, the **ITC** is favored because of the greater σ bond strength of C-N in the product (vide supra). The explanation of the slower rate reaction of [3]-rearrangement of **ITC**-**TC** pairs is poor overlap of frontier orbitals in the transition state (row 2 versus row 3 element). At equilibrium in cyclohexane, allyl thiocyanate is undetectable, but in acetonitrile the mol fraction becomes 9–11%, a result consistent with the higher dipole moment of allyl isothiocyanate and an ionic component (dissociative) to the transition state [29]. 

### 2.3. Adventitious Capture of NCS^−^

**ITC**s are expressed by bacteria from isonitriles by task-specific adapted rhodanese. Rhodanese is distributed widely in the Nature and carries out the important role of scavenging fugitive inorganic cyanide formed during metabolism, e.g., ‘leakage’ from C-1 tetrahydrofolate metabolism or other cyanide-generating reactions [31,32,33]. A key intermediate, cyanoformate (NC-COOH, **CF**), is formed from 1-aminocyclopropane-1-carboxylate (oxidation product of Gly) in the biosynthesis of the plant hormone ethylene (CH_2_=CH_2_) [34]. In *Burkholderia gladioli* that produces a non-terpenoid isonitrile, Hertwick and coworkers showed that rhodanese RhDE, in addition to donating competency to scavenge cyanide NC^−^ into thiocyanate, NCS^−^, catalyzes the substrate-specific sulfur transfer reaction R-NC → R-NCS generating an **ITC**, sinapigladioside [35], by utilizing thiosulfate as an S donor [36]. Hertwick speculates that the enzyme was recruited from a ‘ubiquitous detoxification enzyme for the formation of a bioactive specialized metabolite’ [36]. The concept of the bacterial recruitment of rhodanese, with evolved substrate specificity to the latter task, may also find support in the biosynthesis of terpenoid **ITC**s in sponges and the observation that **TI**s are not uniformly accompanied by their **ITC** counterparts. While rhodanese is ubiquitous across the Kingdoms of Life, no reports of its specific occurrence in Porifera have appeared yet.

### 2.4. Alkyl TC from S_N_ Reaction with Kinetically Competent NCS^−^

Pearson and coworkers compiled the relative rates of nucleophilic substitutions of methyl iodide, CH_3_I, with selected nucleophiles under comparable conditions (25 °C, Table 1) [37]. The second-order rate constant values, *k*_2_, for the reaction with NCS^−^ and NC^−^ are similar (Entries 8 and 9: 5.74 × 10^−4^ and 6.5 × 10^−5^ mol^−1^·s^−1^, respectively (for comparison, the *k*_2_ values for the reactions with thiophenoxide, PhS^−^, and thiosulfate, S_2_O_3_^2–^, are 1.07 and 0.114 mol^−1^·s^−1^ [37]).

**Scheme 1:**
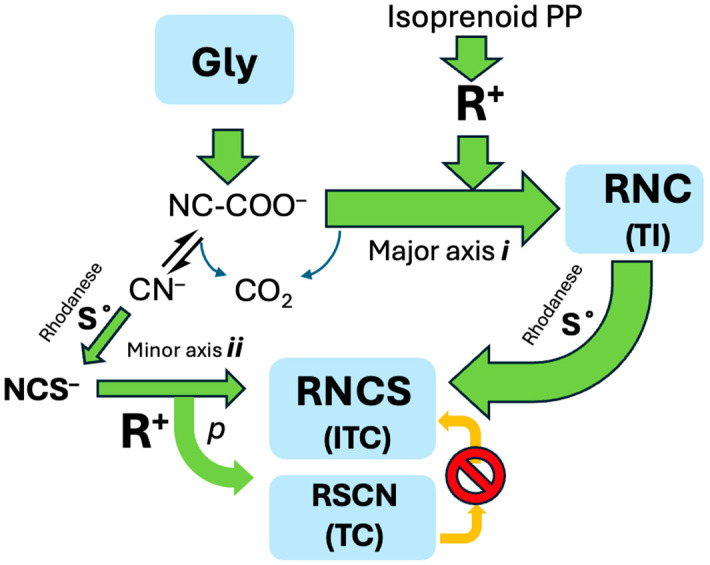
Proposal for the biosynthesis of sponge terpenyl isonitrile (**TI**), isothiocyanate (RNCS, **ITC**), and thiocyanate (RSCN, **TC**) natural products along two axes: major ***i*** and minor ***ii***. *p* is the partition ratio of **ITC:TC** along ***ii***.

A simplified scenario can be proposed for the terminal step in **TC** and **ITC** biosynthesis (Scheme 1). The *S*_N_1 capture of NCS^−^ at S (the more nucleophilic end) yields **TC**s (*minor axis*). **ITC**s formed in this way could contribute to the pool of **ITC**s generated by the S° addition to the corresponding **TI**s. Experimentally, it is conceivable that the fraction of **ITC**s that arises from *minor* and *major axes* could be estimated from isotopic labeling (see below).

Inspection of the structures of secondary metabolites containing the R-SCN group (**TC**s) precludes an enzyme system for the tailoring of R-NCS to R-SCN, but can support a role of rhodanese as a generator of the ambident nucleophile, NCS^−^. **ITC**s may also arise from the less-favored capture of NCS^−^ at N by *S*_N_1 addition, a reaction that would add to the pool of **ITC**s arising from the *major axis* (Scheme 1).

In the laboratory, mixtures of **TC**s and **ITC**s are formed from the reaction of NCS^−^ with electrophiles, e.g., R-Br. In contrast, the replacement of NCS^−^ with NC^−^ in *S*_N_ substitution reactions yields only nitriles, R-CN, not isonitriles, RNC, by an exclusive nucleophilic addition at C [9]. At equilibrium, isomerization would favor **ITCs** over **TCs** based on the relative bond strengths of C-N compared with C-S (see Supporting Information), but this is kinetically prohibitive.

### 2.5. A Simple Proposal—Thiocyanates Can Arise Adventitiously

How do these foregoing data affect the interpretation of the provenance and distribution of alkyl isothiocyanate, **ITC**, versus alkyl thiocyanate, **TC**? Alkyl **TC**s are rarer than their isomeric **ITC**s. Two pathways or axes for the generation of S isonitriloids can be proposed (Scheme 1). Biosynthetically, **ITC**s arise mostly from the linear pathway (major axis, ***i***) of **TI** → **ITC** by the formal addition of S° to the terminus of R-NC from a suitable donor. The less abundant **TC**s more likely arise from free thiocyanate, NCS^−^ (minor axis, ***ii***), delivered by rhodanese-promoted capture of fugitive cyanide, and its nucleophilic addition to the terpenoid carbocation, R^+^ (see Supporting Information for expanded Scheme S1). The partition ratio, *p* (**TC**:**ITC**), along the *minor axis **ii*** may be measurable by {^14^C}-NCS^−^ labeling in a suitable sponge candidate known to produce both isomeric **ITC**s and **TC**s. **TC**s would be expected to predominate over **ITC**s (*p* > 1) in ***ii***, but, overall, ***i*** dominates the production of **ITC**s.

**Scheme 2:**
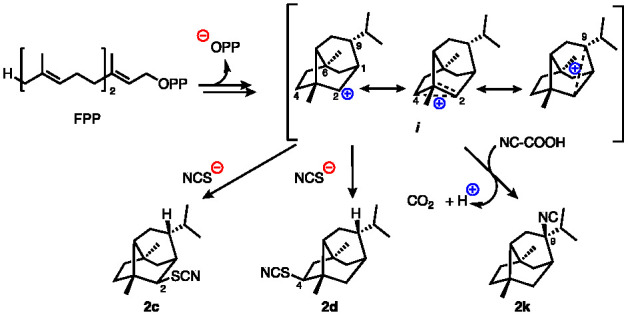
A unifying proposal for the biosynthesis of **2c**, **d**, **k** with the intermediate non-classical cation ***i***.

Scheme 1 leads to a unifying biosynthetic proposal for the biogenesis of **TC**, **ITC** and **TI** isonitriloids; for example, the three iso-morphic neopupukeanane natural products, **2c**, **d**, **k** (Scheme 2), link thiocyanate and isonitrile formation. The ionization of farnesyl pyrophosphate (FPP), followed by stepwise cyclizations, as first proposed by Scheuer [15] and Opatz [1] and respective coauthors, yields stabilized incipient carbocation, ***i*** (represented in this paper by non-classical resonance forms [24,38]), which is captured by a C_1_ nucleophile (NCS^−^ or NC-COOH) to yield the isonitriloids **2c**, **d**, **k**. While the absolute configuration (AC) of neopupukeanane isonitriloids have not been independently assigned, **2c**, **d**, **k** as depicted here conform to the rational biosynthetic proposal by Scheuer from amorphanyl cation [15] and his original assignment of the AC of the pupukeanane skeleton by CD [13].

One question remains: ‘how is exogenous NCS^−^ assimilated by sponges that make **TC**-**ITC** pairs?’ HCN is a weak Brønsted acid (p*K*a = 9.25), but HSCN is strong (p*K*a = −0.7 [39] and fully ionized at physiological pH values. While exogenous cyanide can intercept the intracellular dynamic equilibrium of cyanoformate (CF, NC-COOH) by passive diffusion in its neutral form, HCN, the p*K*_a_ of HSCN would seem prohibitively low for a similar process; a ‘thiocyanate ion transporter’ would need to be invoked [40]. Yet, there is precedence. *Thiohalobacter* spp., with induced capacity to metabolize NCS^−^, have been raised by repeated passage in culture media containing NCS^−^ [41].

It is notable that, in the **TI**-**ITC**-**TC**-producing sponges, capture by H_2_O is rarely or never observed (the absence of corresponding alcohols, R-OH, or alkene elimination products), suggesting a tight coupling of terpenoid–NC capture, possibly in a reaction within an enzyme active site where H_2_O molecules are excluded. The evaluation of this proposal must await a missing dataset: the first high-resolution X-ray or cryo-EM structure of an isonitriloid terpenoid cyclase.

Allylic thiocyanate, **8c**, and isothiocyanate, **8b** (Figure 1), hypothetically obtainable from the nucleophilic substitution of farnesyl pyrophosphate, or **8b** separately from S-transfer to farnesyl isonitrile (**8a**) (which, itself, ‘remains elusive’ [1]), are kinetically labile. **TC**s, in time, would be expected to isomerize by allylic rearrangement to their more substituted *tert*-NCS counterparts (e.g., **9**). Most sponge-derived **TC**s, however, are ‘fixed’, stabilized by substitution at 2° or 3° alkyl groups. Farnesyl isothiocyanate (**8b**) [42] and formamide, **8d**, are known natural products [43], but the foregoing reasons make it unlikely that the ‘missing’ **8c** will ever be isolated from natural sources, as [3] rearrangement irreversibly converts **8c** to its isomeric nerolidyl isothiocyanate isomer, **9**. The fact that **9** has not been found in Nature suggests that **8c**, too, is absent. The syntheses of **8c** and its geranyl homolog were found as mixtures of **8c** and the [3] rearrangement product, **9** (**8c**:**9**~83:17), even after mild vacuum distillation conditions [44]. Although allyl derivatives **6** and **7** exhibit good antibacterial properties, their geranyl and farnesyl analogs (e.g., **8b**,**c**) showed none. It should be noted that the names ‘stylotellane A’ and ‘stylotellane B’ were conferred upon the carbonimidic dichloride **10** and related **11** [11] from *Styletta aurantium* [40]. Compound **11** was originally isolated by Wratten and Faulkner from *Pseudaxinyssa pitys*—the first example of this functional group in a natural product [11].

Compounds **8b**,**d** and **10** complete the set of known farnesyl N-derivatives. It appears that **2m** is the only allylic **TC** among these marine natural products, with the SCN group substituted at the ‘tail’ of the first isoprene group in the precursor FPP. The unexpected, unbranched long-chain **ITC**s, **12**, from *Pseudoaxinyssa*, contain vinyl-substituted α,ω-bisisocyanato groups and appear to have different, indeterminate biosyntheses [45].

One consequence of a strictly unimolecular rate law of reaction of NCS^−^ with chiral R^+^ is diastereomeric mixtures. The product distribution will be dependent upon the usual stereoelectronic factors that govern the stereofacial preferences of *S*_N_1 reactions. For example, in the case of epimeric **2g** and **2h**, isolated from the sponge *Axinyssa aculeata* [20], the reported ratio is 3:2 [46], but, from local symmetry considerations, there would be little or no stereofacial preference for attack on the incipient carbocation. In *Phyllidia varicosa*, the nudibranch that depredates *A. aculeata* and sequesters **2g** and **2h**, the epimer ratio is altered: in the dorsal mantle it is 1:2, but about equimolar in the digestive organ. Fractionation of secondary metabolites by nudibranchs from their sponge diets, as well as metabolic modification, have both been described before, e.g., trisoxazole macrolides from the Spanish Dancer, *Hexbranchus sangineus* [46].

When thought of in this way, the capture of carbocations by free NCS^−^ is a ‘clock reaction’, a kinetic monitor of the partition between the shunt reactions of cyanoformate, **CF** (NC-COO^−^ Scheme 1 and Scheme 2)—with dissociation to NC^−^ and CO_2_—and the direct nucleophilic *S*_N_1 capture by NCS^−^. Garson [12,47] and others [48] have shown through numerous radiolabeling experiments that inorganic [^14^C]-CN^−^ is assimilated into sponge **TI**s. For example, 7,20-diisocyanoadociane (**3**) is radiolabeled by [^14^C]-CN^−^ [49]. These observations have recently been interpreted as an interception of an equilibrium cyanide pool formed by the dissociation of **CF**, the putative ‘NC’ vector, and exogenous uptaken HCN [9]. In the same study, Garson found no radiolabel incorporation into **3** when the sponge was incubated with [2-^14^C] Gly or a number of other amino acids [50]. It is felt that a lack of detection of radiolabeled **3** after incubation with [2-^14^C]Gly is not paradoxical, but rather a consequence of below-threshold incorporation and much faster assimilation into sponge tissue protein and other intermediary metabolism.

The prevailing belief to date was that inorganic NC^−^ is the precursor of **TI**s, but, for several reasons, this was shown to be untenable [9]. If it were so, a simple test can be made: the expected labeling of living sponges with [^14^C]-CN^−^ should also induce the formation of limited amounts of R-SCN (from rhodanese-generated NCS^−^-favored *S*_N_1 nucleophile capture at S), but this has not been observed to date. A counter explanation would be that fraction of exogenous cyanide that is not incorporated into the structures of sponge **TI**s is rapidly and efficiently converted to NCS^−^ by rhodanese in separate cellular compartments, spatially distal from the active sites of **TI** biosynthetic enzymes and becomes unavailable for **TC** or **ITC** production. Garson and coworkers showed that radiolabeled thiocyanate (K [^14^C]-NCS) is assimilated by living sponges into **TI**s, including axisonitrile-3 (notably with a formula lacking S), and two **ITC**s (axisothiocyanate-3), albeit with far lower specific activities than the same radiolabeled natural products obtained from incubations with Na{^14^C]-CN [51]. The simultaneous labeling of axisonitrile-3 from the incubation of the sponge *Acanthella cavernosa* [52] with K [^14^C]-NCS requires, at the very least, an unspecified interchange of thiocyanate with K [^14^C]-CN (e.g., it is known mammalian oxyhemoglobin can convert thiocyanate to cyanide [53]). In a separate study, Simpson and Garson found the incorporation of both [^14^C]-SCN^−^ and [^14^C]-CN^−^ into the sesquiterpene thiocyanato group of **2b** [21], produced by *Amphimedon terpenensis* from the Great Barrier Reef [54]; both results are consistent with the adventitious thiocyanate model for **TC** biosynthesis (Scheme 1). What could be testable in the field is the incorporation of {2-^14^C}-NC-COOH in sponges producing **TI**s, **ITC**s, and **TC**s or {^14^C}-NCS^−^ under more controlled conditions. The outcomes would be compelling, but the logistics of this proposed experiment are beyond the scope of this work.

## 3. Materials and Methods

### General Experimental Procedures

DFT Calculations. All DFT calculations were performed using Spartan ’20 (Wavefunction, Irvine, CA, USA), using functional ωB97X-D and basis set 6-31G* (H_2_O or gas phase). The coordinates for the optimized structures of **4**, **5**, and **S1** and bond parameters and energies can be found in the Supporting Information. See Supporting Information for a complete citation for the DFT product.

## 4. Conclusions

A unified biosynthetic scheme is proposed to explain the naturally occurring **TI**, **ITC**, and **TC** isonitriloids that engage the ‘NC’ group of cyanoformate (NC-COO^−^). While the major axis of the biosynthesis of **ITC**s involves S° transfer to **TI**s, isomeric **TC**s are outliers on the linear path **IT** → **ITC**. It is proposed **TC**s arise from the adventitious capture of incipient terpenoid carbocation, R^+^, by inorganic NCS^−^, itself delivered by the rhodanese-promoted capture of fugitive NC^−^ by an S° donor.

## Figures and Tables

**Table 1:** Second-order rate constant (*k*_2_, *S*_N_2) of CH_3_-I with the selected nucleophiles (25 °C) (from Pearson [37]).

Entry	Nu: or Nu:^−^	10^3^·*k*_2_/M^−1^·s^−1^	Entry	Nu: or Nu:^−^	10^3^·*k*_2_/M^−1^·s^−1^
1	MeOH	1.3 × 10^−7^	7	PhO^−^	0.073
2	NH_3_	0.041	8	NCS^−^	0.574
3	N_3_^−^	0.078	9	NC^−^	0.645
4	Br^−^	0.0798	10	NCSe^−^	9.13
5	I^−^	3.42	11	PhS^−^	1070
6	(CH_3_)_2_S	0.045	12	S_2_O_3_^2–^	114

## Data Availability

Original data will be made available upon reasonable request.

## Supplementary Materials

The following supporting information can be downloaded at: https://www.mdpi.com/article/10.3390/md23050220/s1. DFT-calculated structures of **4**, **5**, and 1-isothiocyanato-1-methylcyclohexane (**S1**).
