# Supplementary material for: The Enigma of Sponge-Derived Terpenoid Isothiocyanate–Thiocyanate Pairs: A Biosynthetic Proposal
Source: Mar Drugs. 2025 May 21;23(5):220. doi: 10.3390/md23050220 (PMC12112954; doi:10.3390/md23050220)
Supplement: Supplementary file 1 [file marinedrugs-23-00220-s001.zip › marinedrugs-3511805-supplementary.pdf]

Supporting Information

The Enigma of Sponge-Derived Terpenoid Isothiocyanates-Thiocyanates. A Biosynthetic Proposal

Tadeusz F. Molinski[1,2,*]

1 Department of Chemistry and Biochemistry, University of California, San Diego, 9500 Gilman Drive MC3568, La Jolla, CA 92093, USA; tmolinski@ucsd.edu

2 Skaggs School of Pharmacy and Pharmaceutical Sciences, University of California, San Diego, 9500 Gilman Drive, University of California, San Diego, 9500 Gilman Drive, La Jolla, CA 92093, USA.

* Correspondence: tmolinski@ucsd.edu

Page	Title	Content
S1.	**Table S1**	DFT minimized (ωB97X-D 6-31G*) structure of 1-isocyano-1-methylcyclohexane (**S1**).
S2	**Table S2**.	DFT minimized (ωB97X-D 6-31G*) structure of *t*-Bu-SCN (**4**).
S3	**Table S3**	DFT minimized (ωB97X-D 6-31G*) structure of *t*-Bu-NCS (**5**).
S4	**Table S4**	Energy and bond order parameters of the DFT calculated structure of *t*-Bu-SCN (**4**).
S5	**Table S5**	Energy and bond order parameters of the DFT calculated structure of *t*-Bu-NCS (**5**).
S5	**Table S6**	Comparative energies (DFT) of *t*-Bu-SCN (**4**) and *t*-Bu-NCS (**5**
S6	**–**	Citation to Spartan ’20 engine.
S6	**Scheme S1**	Complete unified proposal for biosynthesis of sponge terpene isonitrile (**TI**), isothiocyanate (**ITC**) and thiocyanate (**TC**) natural products.
S7	**Table S7**	Structures of key isonitriloid natural products, **1**-**3**: trivial names and references.

Table S1. Coordinates for the structure of 1-isocyano-1-methylcyclohexane (**S1**).
DFT minimized (ωB97X-D 6-31G*, non-polar solvent).

```
HEADER
REMARK Spartan'20 exported M0001
HETATM    1  H   UNK  0001       1.515  -0.630  -2.331
HETATM    2  C   UNK  0001       1.138  -0.158  -1.418
HETATM    3  C   UNK  0001       1.472   0.074   1.076
HETATM    4  C   UNK  0001      -0.899  -0.036   0.124
HETATM    5  C   UNK  0001       0.025  -0.363   1.312
HETATM    6  C   UNK  0001      -0.309  -0.596  -1.185
HETATM    7  C   UNK  0001       2.026  -0.517  -0.224
HETATM    8  H   UNK  0001       1.171   0.927  -1.587
HETATM    9  H   UNK  0001       2.086  -0.235   1.929
HETATM   10  H   UNK  0001       1.523   1.170   1.031
HETATM   11  H   UNK  0001      -0.382   0.096   2.220
HETATM   12  H   UNK  0001      -0.015  -1.450   1.449
HETATM   13  H   UNK  0001      -0.951  -0.303  -2.023
HETATM   14  H   UNK  0001      -0.355  -1.690  -1.105
HETATM   15  H   UNK  0001       2.077  -1.611  -0.129
HETATM   16  H   UNK  0001       3.049  -0.162  -0.393
HETATM   17  C   UNK  0001      -2.316  -0.562   0.363
HETATM   18  H   UNK  0001      -2.281  -1.651   0.460
HETATM   19  H   UNK  0001      -2.971  -0.304  -0.475
HETATM   20  H   UNK  0001      -2.734  -0.141   1.284
HETATM   21  N   UNK  0001      -0.977   1.402  -0.001
HETATM   22  C   UNK  0001      -0.954   2.567  -0.111
HETATM   23  S   UNK  0001      -0.938   4.176  -0.264
CONECT    1    2
CONECT    2    1    6    7    8
CONECT    3    5    7    9   10
CONECT    4    6    5   17   21
CONECT    5    3    4   11   12
CONECT    6    2    4   13   14
CONECT    7    2    3   15   16
CONECT    8    2
CONECT    9    3
CONECT   10    3
CONECT   11    5
CONECT   12    5
CONECT   13    6
CONECT   14    6
CONECT   15    7
CONECT   16    7
CONECT   17   18   19   20    4
CONECT   18   17
CONECT   19   17
CONECT   20   17
CONECT   21    4   22
CONECT   22   21   23
CONECT   23   22
END
```

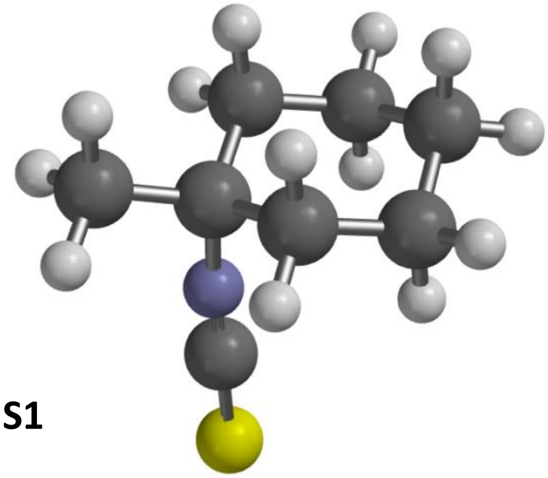

S1

Table S2. DFT minimized (ωB97X-D 6-31G*) structure of *t*-Bu-SCN (**4**).
DFT minimized (ωB97X-D 6-31G*, non-polar solvent).

```
HEADER
REMARK Spartan'20 exported M0001
HETATM    1  C   UNK  0001       -0.216   0.000  -0.127
HETATM    2  S   UNK  0001       -1.293   0.000   1.410
HETATM    3  C   UNK  0001       -1.227   0.000  -1.277
HETATM    4  H   UNK  0001       -1.864  -0.890  -1.255
HETATM    5  H   UNK  0001       -0.680   0.000  -2.225
HETATM    6  H   UNK  0001       -1.864   0.890  -1.255
HETATM    7  C   UNK  0001        0.646   1.262  -0.151
HETATM    8  H   UNK  0001        1.244   1.269  -1.071
HETATM    9  H   UNK  0001        1.334   1.291   0.699
HETATM   10  H   UNK  0001        0.030   2.165  -0.133
HETATM   11  C   UNK  0001        0.646  -1.262  -0.151
HETATM   12  H   UNK  0001        1.244  -1.269  -1.071
HETATM   13  H   UNK  0001        0.030  -2.165  -0.133
HETATM   14  H   UNK  0001        1.334  -1.291   0.699
HETATM   15  C   UNK  0001       -0.094   0.000   2.610
HETATM   16  N   UNK  0001        0.730   0.000   3.430
CONECT    1    2    3    7   11
CONECT    2    1   15
CONECT    3    4    5    6    1
CONECT    4    3
CONECT    5    3
CONECT    6    3
CONECT    7    8    9   10    1
CONECT    8    7
CONECT    9    7
CONECT   10    7
CONECT   11   12   13   14    1
CONECT   12   11
CONECT   13   11
CONECT   14   11
CONECT   15    2   16
CONECT   16   15
END
```

Table S3. DFT minimized (ωB97X-D 6-31G*) structure of *t*-Bu-NCS (**5**).
DFT minimized (ωB97X-D 6-31G*, non-polar solvent).

```
HEADER
REMARK Spartan'20 exported M0001
HETATM    1  C   UNK  0001      -0.042   0.000  -0.086
HETATM    2  C   UNK  0001      -0.883  -1.264  -0.303
HETATM    3  H   UNK  0001      -1.238  -1.281  -1.338
HETATM    4  H   UNK  0001      -1.749  -1.272   0.365
HETATM    5  H   UNK  0001      -0.285  -2.161  -0.122
HETATM    6  C   UNK  0001       1.195   0.000  -0.992
HETATM    7  H   UNK  0001       1.804   0.891  -0.813
HETATM    8  H   UNK  0001       0.871  -0.000  -2.037
HETATM    9  H   UNK  0001       1.804  -0.891  -0.813
HETATM   10  C   UNK  0001      -0.883   1.264  -0.303
HETATM   11  H   UNK  0001      -1.239   1.281  -1.338
HETATM   12  H   UNK  0001      -0.285   2.162  -0.123
HETATM   13  H   UNK  0001      -1.749   1.273   0.365
HETATM   14  N   UNK  0001       0.414   0.000   1.285
HETATM   15  C   UNK  0001       0.845  -0.000   2.372
HETATM   16  S   UNK  0001       1.421  -0.000   3.882
CONECT    1    2    6   10   14
CONECT    2    3    4    5    1
CONECT    3    2
CONECT    4    2
CONECT    5    2
CONECT    6    7    8    9    1
CONECT    7    6
CONECT    8    6
CONECT    9    6
CONECT   10   11   12   13    1
CONECT   11   10
CONECT   12   10
CONECT   13   10
CONECT   14   15    1
CONECT   15   14   16
CONECT   16   15
END
```

Table S4. Energy and bond order parameters of the DFT calculated structure of *t*-Bu-SCN (**4**). E = –648.779796 Hartrees.

▼ Molecular Orbital Energies

Show First 3 LUMOs

Label	Energy (ev)	Sym. Lab.
LUMO{+2}	3.27	A"
LUMO{+1}	3.15	A'
**LUMO**	1.35	A'
**HOMO**	-9.16	A"
HOMO{-1}	-10.68	A'
HOMO{-2}	-11.95	A'
HOMO{-3}	-11.97	A"
HOMO{-4}	-12.21	A'
HOMO{-5}	-12.34	A"
HOMO{-6}	-12.37	A'
HOMO{-7}	-12.82	A"
HOMO{-8}	-13.43	A'
HOMO{-9}	-13.64	A"

▼ Calculated Bond Orders

Bond	Löwdin	Mulliken
**C1 - C2**	1.066	0.990
**C1 - C3**	1.072	0.987
**C1 - C4**	1.072	0.987
**C1 - S1**	0.980	0.919
**C2 - H1**	0.934	0.938
**C2 - H5**	0.931	0.938
**C2 - H6**	0.934	0.938
**C3 - H4**	0.931	0.937
**C3 - H7**	0.930	0.932
**C3 - H8**	0.936	0.942
**C4 - H2**	0.931	0.937
**C4 - H9**	0.936	0.942
**C4 - H10**	0.930	0.932
**C5 - N1**	3.086	2.677
**C5 - S1**	1.259	1.059

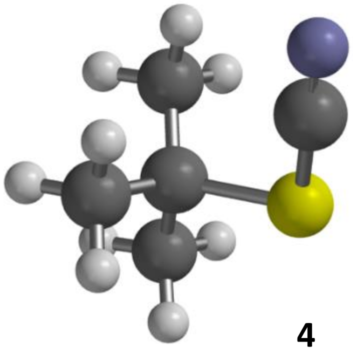

4

**Name:**	M0001
**Formula:**	C_5H_9NS

**Job type:**	Equilibrium Geometry
**Method:**	ωB97X-D
**Basis set:**	6-31G*
**Energy:**	-648.779796 hartrees

Table S5. Energy and bond order parameters of the DFT calculated structure of *t*-Bu-NCS (**5**).
E = –648.803309 Hartrees.

Molecular Orbital Energies

Show First 3 LUMOs

Label	Energy (ev)
LUMO{+2}	4.18
LUMO{+1}	2.32
**LUMO**	2.30
**HOMO**	-8.78
HOMO{-1}	-8.78
HOMO{-2}	-11.60
HOMO{-3}	-11.60
HOMO{-4}	-12.42
HOMO{-5}	-12.67
HOMO{-6}	-12.67
HOMO{-7}	-13.01
HOMO{-8}	-13.68
HOMO{-9}	-13.69

Calculated Bond Orders

Bond	Löwdin	Mulliken
C1 - C2	1.048	0.993
C1 - C3	1.047	0.993
C1 - C4	1.048	0.993
C1 - N1	0.991	0.840
C2 - H4	0.931	0.934
C2 - H5	0.936	0.937
C2 - H6	0.936	0.937
C3 - H1	0.936	0.937
C3 - H7	0.932	0.934
C3 - H8	0.936	0.937
C4 - H2	0.931	0.934
C4 - H9	0.936	0.937
C4 - H10	0.936	0.937
C5 - N1	2.457	2.162
C5 - S1	1.787	1.558
N1 - S1	0.347	

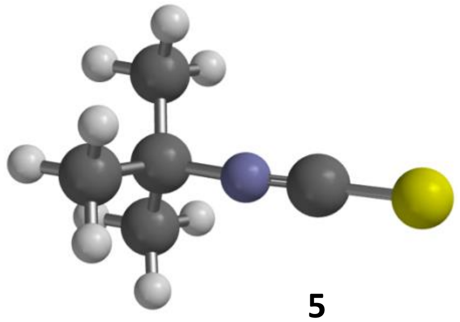

5

**Name:**	M0001
**Formula:**	C_5H_9NS

**Job type:**	Equilibrium Geometry
**Solvent:**	Nonpolar
**Method:**	ωB97X-D
**Basis set:**	6-31G*
**Energy:**	-648.803309 hartrees

Table S6. Comparative energies (DFT) of *t*-Bu-SCN (**4**) and *t*-Bu-NCS (**5.**)
E = –648.779796 Hartrees.

Cmpd.	E_h (Hartrees)	E_h – 648	ΔE_h	ΔE /kcal.mol^{-1}.
**4**	–648.779796	0.779796	0.000000	0.000000
**5**	–648.803309	0.803309	-0.023513	-14.75464263

Cmpd.	E_h (Hartrees)	E_h – 648	ΔE_h	ΔE /kcal.mol^{-1}.
**4**	–648.779796	0.779796	0.000000	0.000000
**5**	–648.796485	0.796485	-0.016689	-10.47251439

Scheme S1. Complete unified proposal for biosynthesis of sponge terpene isonitrile (**TI**), isothiocyanate (**ITC**) and thiocyanate (**TC**) natural products. See also Reference xx.

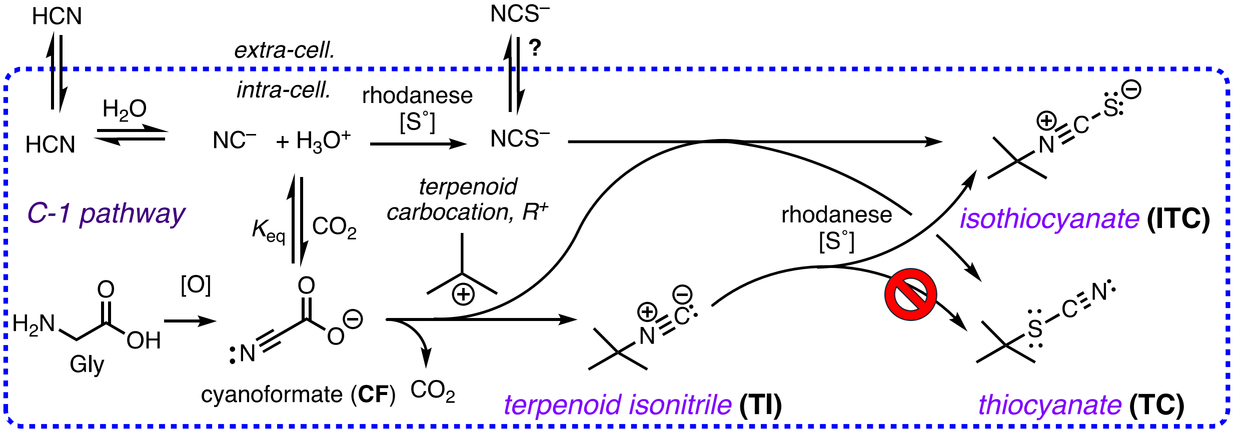

Spartan Citation.

Yihan Shao, Zhengting Gan, E. Epifanovsky, A. T. B. Gilbert, M. Wormit,, J. Kussmann, A. W. Lange, A. Behn, Jia Deng, Xintian Feng, D. Ghosh,, M. Goldey, P. R. Horn, L. D. Jacobson, I. Kaliman, T. Kus, A. Landau, Jie Liu,, E. I. Proynov, R. M. Richard, R. P. Steele, E. J. Sundstrom,, H. L. Woodcock III, P. M. Zimmerman, D. Zuev, B. Alam, B. Albrecht,, E. Alguire, S. A. Baeppler, D. Barton, Z. Benda, Y. A. Bernard,, E. J. Berquist, K. B. Bravaya, H. Burton, K. Carter-Fenk, D. Casanova,, Chun-Min Chang, Yunqing Chen, A. Chien, K. D. Closser, M. P. Coons,, S. Coriani, S. Dasgupta, A. L. Dempwolff, M. Diedenhofen, Hainam Do,, R. G. Edgar, Po-Tung Fang, S. Faraji, S. Fatehi, Qingguo Feng, J. Fosso-Tande,, J. Gayvert, Qinghui Ge, A. Ghysels, G. Gidofalvi, J. Gomes, J. Gonthier,, A. Gunina, D. Hait, M. W. D. Hanson-Heine, P. H. P. Harbach, A. W. Hauser,, M. F. Herbst, J. E. Herr, E. G. Hohenstein, Z. C. Holden, Kerwin Hui,, B. C. Huynh, T.-C. Jagau, Hyunjun Ji, B. Kaduk, K. Khistyaev, Jaehoon Kim,, P. Klunzinger, K. Koh, D. Kosenkov, L. Koulias, T. Kowalczyk, C. M. Krauter,, A. Kunitsa, Ka Un Lao, A. Laurent, K. V. Lawler, Joonho Lee, D. Lefrancois,, S. Lehtola, D. S. Levine, Yi-Pei Li, You-Sheng Lin, Fenglai Liu, Kuan-Yu Liu,, E. Livshits, M. Loipersberger, A. Luenser, P. Manohar, E. Mansoor,, S. F. Manzer, Shan-Ping Mao, Yuezhi Mao, N. Mardirossian, A. V. Marenich,, T. Markovich, L. A. Martinez-Martinez, S. A. Maurer, N. J. Mayhall,, S. C. McKenzie, J.-M. Mewes, P. Morgante, A. F. Morrison, J. W. Mullinax,, K. Nanda, T. S. Nguyen-Beck, R. Olivares-Amaya, J. A. Parkhill, S. K. Paul,, Zheng Pei, T. M. Perrine, F. Plasser, P. Pokhilko, S. Prager, A. Prociuk,, E. Ramos, B. Rana, D. R. Rehn, F. Rob, M. Scheurer, M. Schneider, N. Sergueev,, S. M. Sharada, S. Sharma, D. W. Small, T. Stauch, C. J. Stein, T. Stein,, Yu-Chuan Su, S. P. Veccham, A. J. W. Thom, A. Tkatchenko, T. Tsuchimochi,, N. M. Tubman, L. Vogt, M. L. Vidal, O. Vydrov, M. A. Watson, J. Wenzel,, M. de Wergifosse, T. A. Wesolowski, A. White, J. Witte, A. Yamada, Jun Yang,, K. Yao, S. Yeganeh, S. R. Yost, Zhi-Qiang You, A. Zech, Igor Ying Zhang,, Xing Zhang, Yan Zhao, Ying Zhu, B. R. Brooks, G. K. L. Chan, C. J. Cramer,, M. S. Gordon, W. J. Hehre, A. Klamt, M. W. Schmidt, C. D. Sherrill,, D. G. Truhlar, A. Aspuru-Guzik, R. Baer, A. T. Bell, N. A. Besley,, Jeng-Da Chai, A. E. DePrince, III, R. A. DiStasio Jr., A. Dreuw,, B. D. Dunietz, T. R. Furlani, Chao-Ping Hsu, Yousung Jung, Jing Kong,, D. S. Lambrecht, WanZhen Liang, C. Ochsenfeld, V. A. Rassolov,, L. V. Slipchenko, J. E. Subotnik, T. Van Voorhis, J. M. Herbert, A. I. Krylov,, P. M. W. Gill, M. Head-Gordon. Advances in molecular quantum chemistry contained in the Q-Chem 4 program package. *Mol. Phys.* **2015**, *113*, 184-215.

Table S7. Structures of key isonitriloid natural products, **1-3**: trivial names and references.[1]

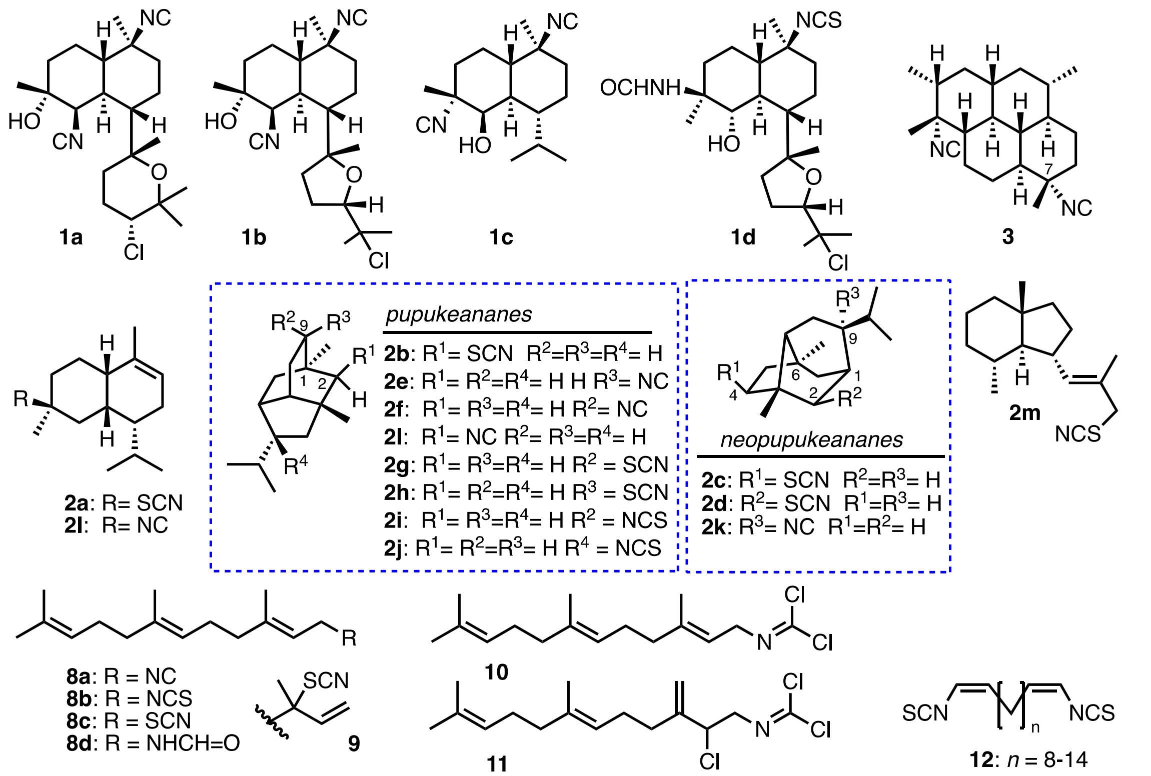

#	#[a]	Compound	Ref.	Author, journal.
**1a**	Kol1	Kalihinol A	[2]	Scheuer, Clardy, et al. *JACS* **1984**
**1b**	Kol20	Kalihinol B	[3]	Baker, Scheuer, Clardy, et al. *JACS* **1984**
**1c**	–	MED6-189	[4]	Chahine, Z, et al. *Science* **2024**
**1d**	Kol39	Kalihinol M	[5]	Xu, Y. S. China Sea. *Tetrahedron* **2012**
**2a**	Ca20	thiocyanatocadinene	[6]	He & Faulkner, *JOC* **1989**
**2b**	Pu-7	2-isocyanopupukeanane	[7]	Scheuer, *Helv. Chim. Acta*. **1979**
**2c**	Pu13	4-thiocyanato*neo*pupukeanane	[8]	Pham, Fusetani, *Tet. Lett*. **1991**
**2d**	Pu12	2-thiocyanato*neo*pupukeanane	[9]	He, H. & Faulkner, D.J. et al. *JOC* **1992**
**2e**	Pu2	9-isocyanopupukeanane	[10]	Burreson, Scheuer, Clardy, *JACS* **1975**
**2f**	Pu3	9-*epi*-isocyanopupukeanane	[11]	Fusetani, *Tet. Lett.***1990**.
**2g**	Pu5	9-thiocyanatopupukeanane	[12]	Proksch, et al. *J. Nat. Prod*. **2003**
**2h**	Pu6	*epi*-9-thiocyanatopupukeanane	[12]	Proksch, et al. *J. Nat. Prod*. **2003**
**2i**	Pu4	9-isothiocyanatopupukeanane	[13]	Simpson, Garson, *Aust. J. Chem*. **1997**
**2j**	Pu10	5-isothiocyanatopupukeanane	[14]	Marcus, et al. *JOC* **1989**
**2k**	Pu11	9-isocyano*neo*pupukeanane	[15]	Karuso & Scheuer, *JOC* **1989**
**2l**	Ca12	10α-isocyano-4-amorphene	[16]	Fusetani et al, *Tet. Lett.* **1992**.
**2m**	Ax9	(–)-Cavernothiocyanate (allylic R-SCN)	[16]	Fusetani et al, *Tet. Lett.* **1992**.
**3**		1,7-Diisocyanoadociane	[17]	Baker, et al. *JACS* **1976**

[a]Cross-reference numbering to the Opatz review.[18]

[1] The absolute configurations of the compounds are mostly not confirmed.

[2] Chang, W.J.; Patra, A.; Roll, D.M.; Scheuer, P.J. Matsumoto, G.K.; Clardy, J. Kalihinol-A, a highly functionalized diisocyano diterpenoid antibiotic from a sponge. *J. Am. Chem. Soc.* **1984**, *106*, 4644-4646.

[3] Patra, A.; Chang, C. W. J.; Scheuer, P. J.; Van Duyne, G. D.; Matsumoto, G. K; Clardy, J. An Unprecedented Triisocyano Diterpenoid Antibiotic from a Sponge. *J. Am. Chem. Soc.* **1984**, *106*, 7981-7983

[4] Chahine, Z.; Abel, S.; Hollin, T.; Barnes, G.L.; Chung, J.H.; Daub, M.E.; Renard, I.; Choi, J.Y.; Vydyam, P.; Pal, A.; Alba-Argomaniz, M.; Banks, C.A.S.; Kirkwood, J.; Saraf, A.; Camino, I.; Castenada, P.; Cuevas, J.C.; De Mercado-Arnanz, J.; Fernandez-Alvaro, E.; Garcia-Perez, A.; Ibarz, N.; Viera-Morilla, S.; Prudhomme, J.; Joyner, C.J.; Bei, A.K.; Florens, L.; Ben-Mamoun, C.; Vanderwal, C.D.; Le Roch, K.G. A kalihinol analog disrupts apicoplast function and vesicular trafficking in *P. falciparum* malaria. *Science* **2024**, *385,* 7966 – 7978.

[5] Xu, Y.; Li, N.; Jiao, W-H.; Wang,R-P.; Peng, Y.; Qi, S-H.; Song, S-J.; Chen, W-S.; Lin, H-W. Antifouling and cytotoxic constituents from the South China Sea sponge *Acanthella cavernosa*. *Tetrahedron* **2012**, *68*, 2876–2833.

[6] He, H.Y.; Faulkner, D.J.; Shumsky, J.S.; Hong, K.; Clardy, J. A sesquiterpene thiocyanate and three sesquiterpene isothiocyanates from the sponge *Trachyopsis aplysinoides*. *J. Org. Chem.* **1989**, *54*, 2511–2514.

[7] Hagadone, M.R.; Burreson, B.J.; Scheuer, P.J.; Finer, J.S.; Clardy, J. Defense Allomones of the Nudibranch *Phyllidia varicosa* Lamarck 1801. *Helv. Chim. Acta* **1979**, *62*, 2484–2494.

[8] Pham, A.T.; Ichiba, T.; Yoshida, W.Y.; Scheuer, P.J.; Uchida, T.; Tanaka, J.-I.; Higa, T. Two marine sesquiterpene thiocyanates. *Tetrahedron Lett*. **1991**, *32*, 4843–4846.

[9] He, H.; Salvá, J.; Caíalos, R.F.; Faulkner, D.J. Sesquiterpene Thiocyanates and Isothiocyanates from *Axinyssa aplysinoides*. *J. Org. Chem.* **1992**, *57*, 3191-3194.

[10] Burreson, B.J.; Scheuer, P.J.; Finer, J.; Clardy, J. 9-Isocyanopupukeanane, a marine invertebrate allomone with a new sesquiterpene skeleton. *J. Am. Chem. Soc.* **1975**, *97*, 4763-4764.

[11] Fusetani, N.; Wolstenholme, H.J.; Matsunaga, S. Co-occurrence of 9-isocyanopupukeanane and its C-9 epimer in the nudibranch *Phyllidia bourguini*. *Tetrahedron Lett.* **1990**, *31*, 5623–5624.

[12] Yasman, Y.; Edrada, R.A.; Wray, V.; Proksch, P. New 9-Thiocyanatopupukeanane Sesquiterpenes from the Nudibranch *Phyllidia varicosa* and Its Sponge-Prey *Axinyssa aculeata. J. Nat. Prod.* **2003**, *66*, 1512–1514.

[13] Simpson, J.S.; Garson, M.J.; Hooper, J.N.A.; Cline, E.I.; Angerhofer, C.K. Terpene Metabolites from the Tropical Marine Sponge *Axinyssa* sp. nov. *Aust. J. Chem*. **1997**, 50, 1123–1128.

[14] Marcus, A.H.; Molinski, T.F.; Fahy, E.; Faulkner, D.J.; Xu, C.; Clardy, J. 5-Isothiocyanatopupukeanane from a sponge of the genus *Axinyssa*. *J. Org. Chem*. **1989**, *54*, 5184–5186.

[15] Karuso, P.; Poiner, A.; Scheuer, P.J. Isocyanoneopupukeanane, a new tricyclic sesquiterpene from a sponge. *J. Org. Chem*. **1989**, *54*, 2095–2097.

[16] Fusetani, N.; Wolstenholme, H.J.; Shinoda, K.; Asai, N.; Matsunaga, S.; Onuki, H.; Hirota, H. Two sesquiterpene isocyanides and a sesquiterpene thiocyanate from the marine sponge *Acanthella* cf. *cavernosa* and the Nudibranch *Phyllidia ocellata*. *Tetrahedron Lett.* **1992**, *33*, 6823–6826.

[17] Baker, J. T.; Wells, R. J.; Oberhänsli, W. E.; Hawes, G. B. A New Diisocyanide of Novel Ring Structure from a Sponge. *J. Am. Chem. Soc*. **1976**, *98*, 4010−4012.

[18] Emsermann, J.; Kauhl, U.; Opatz, T. Marine Isonitriles and Their Related Compounds. *Mar. Drugs* **2016**, *14*, 16–83.
